# Supplementary material for: Radiofrequency Ablation of Painful Spinal Metastasis: A Systematic Review
Source: Curr Oncol. 2025 May 23;32(6):301. doi: 10.3390/curroncol32060301 (PMC12192184; doi:10.3390/curroncol32060301)
Supplement: Supplementary file 1 [file curroncol-32-00301-s001.zip › curroncol-3578377-supplementary.pdf]

	Pre-intervention				24-72h post-intervention				2-4 weeks post-intervention				12 weeks post-intervention				24 weeks post-intervention			
Authors	Mean	95% C.I.	S.E.	Z-value	Mean	95% C.I.	S.E.	Z-value	Mean	95% C.I.	S.E.	Z-value	Mean	95% C.I.	S.E.	Z-value	Mean	95% C.I.	S.E.	Z-value
Madaelil TP et al. [12]	8.00	-	-	-	-	-	-	-	3.00	-	-	-	-	-	-	-	-	-	-	-
Greenwood TJ et al. [13]	8.00	7.25-8.75	0.38	20.87	-	-	-	-	2.9	1.822-3.978	0.55	5.273	-	-	-	-	-	-	-	-
Prezzano KM et al. [14]	4.52	3.38-5.66	0.58	7.791	-	-	-	-	2.7	1.626-3.774	0.548	4.927	1.6	0.637-2.563	0.491	3.256	-	-	-	-
Wallace AN et al. [15]	8.00	7.56-8.44	0.22	35.728	-	-	-	-	2.9	2.207-3.593	0.354	8.202	-	-	-	-	-	-	-	-
Tomasian A et al. [16]	8.00	7.88-8.12	0.06	130.476	-	-	-	-	3.00	2.88-3.12	0.061	48.929	3.00	2.88-3.12	0.061	48.929	-	-	-	-
Levy J et al. [17]	8.20	7.91-8.49	0.15	55.836	-	-	-	-	3.9	3.392-4.408	0.259	15.049	3.7	3.209-4.191	0.251	14.769	3.5	2.958-4.042	0.276	12.661
Ragheb A et al. [18]	6.90	-	-	-	3.50	-	-	-	2.80	-	-	-	-	-	-	-	0.70	-	-	-
Bagla S et al. [19]	5.90	-	-	-	-	-	-	-	-	-	-	-	2.1	-	-	-	-	-	-	-
Sayed D et al. [20]	5.77	-	-	-	-	-	-	-	3.33	-	-	-	2.64	-	-	-	2.61	-	-	-
Levy J et al. [21]	7.80	7.57-8.03	0.12	65.854	-	-	-	-	4.7	4.318-5.082	0.195	24.092	3.6	3.19-4.01	0.209	17.223	3.2	2.79-3.61	0.209	15.31
Reyes M et al. [22]	7.90	7.32-8.48	0.30	26.813	3.5	2.899-4.101	0.306	11.422	-	-	-	-	-	-	-	-	-	-	-	-
Anchala PR et al. [23]	7.51	7.08-7.94	0.22	34.539	-	-	-	-	2.25	1.827-2.673	0.216	10.433	-	-	-	-	1.75	1.296-2.204	0.232	7.557
Lane MD et al. [24]	7.20	6.64-7.76	0.29	25.08	-	-	-	-	1.68	-	-	-	-	-	-	-	-	-	-	-
Senol N el al. [25]	7.40	-	-	-	2.50	-	-	-	2.50	-	-	-	-	-	-	-	3.20	-	-	-
Pusceddu C et al. [26]	5.70	5.21-6.19	0.25	22.811	-	-	-	-	0.9	0.686-1.114	0.109	8.233	-	-	-	-	3.00	2.878-3.122	0.062	48.023
Sandri A et al. [27]	8.00	-	-	-	1.80	-	-	-	1.90	-	-	-	-	-	-	-	-	-	-	-
Maugeri R et al. [28]	8.50	-	-	-	3.50	-	-	-	2.80	-	-	-	2.60	-	-	-	3.00	-	-	-
Giammalva GR et al. [29]	7.81	-	-	-	5.20	-	-	-	4.10	-	-	-	3.50	-	-	-	-	-	-	-
Masala S et al. [30]	8.60	-	-	-	2.60	-	-	-	-	-	-	-	-	-	-	-	-	-	-	-
Alfonso M et al. [31]	7.70	6.97-8.43	0.37	20.664	-	-	-	-	2.6	1.985-3.215	0.314	8.286	-	-	-	-	-	-	-	-
Hoffmann RT et al. [32]	8.50	-	-	-	5.50	-	-	-	-	-	-	-	3.50	-	-	-	-	-	-	-
Shawky Abdelgawaad A et al. [33]	7.20	6.68-7.72	0.27	27.11	-	-	-	-	-	-	-	-	3.00	2.525-3.475	0.242	12.372	-	-	-	-
Pusceddu C el al. [34]	8.00	-	-	-	-	-	-	-	0.5	-	-	-	0	-	-	-	-	-	-	-
Madani K et al. [35]	7.30	6.34-8.26	0.49	14.901	-	-	-	-	2.00	0.00	-	-	-	-	-	-	-	-	-	-
Lv N et al. [36]	7.52	7.11-7.93	0.21	35.802	2.79	2.638-2.942	0.773	36.089	2.14	2.026-2.254	0.058	36.678	-	-	-	-	2.23	2.098-2.362	0.067	33.235
Zhou X et al. [37]	7.60	5.82-9.38	0.91	8.386	3.2	1.8-4.6	0.714	4.479	3.5	2.156-4.844	0.686	5.103	-	-	-	-	-	-	-	-
Zheng L et al. [38]	7.69	7.33-8.05	0.18	42.325	6.62	6.296-6.944	0.165	40.008	3.62	3.308-3.932	0.159	22.771	2.77	2.509-3.031	0.133	20.824	2.96	2.667-3.253	0.149	19.833
Wang F et al. [39]	8.46	8.06-8.86	0.21	41.037	1.73	1.269-2.191	0.235	7.354	2.24	1.936-2.544	0.155	14.431	1.83	1.488-2.172	0.175	10.48	1.86	1.489-2.231	0.189	9.832
Han X et al. [40]	7.39	6.83-7.95	0.29	25.869	-	-	-	-	4.52	3.882-5.158	0.325	13.896	-	-	-	-	2.3	1.622-2.978	0.346	6.645
Zhang C et al. [41]	7.86	7.43-8.30	0.22	35.397	-	-	-	-	-	-	-	-	3.51	2.842-4.178	0.341	10.299	-	-	-	-
Tian QH et al. [42]	7.43	7.00-7.86	0.22	34.013	2.25	1.879-2.621	0.189	11.902	1.96	1.568-2.352	0.2	9.788	1.91	1.529-2.291	0.195	9.813	1.86	1.484-2.236	0.192	9.696
He Y et al. [43]	7.19	6.259-8.121	0.475	15.14	4.39	3.787-4.993	0.307	14.28	2.89	2.274-3.506	0.314	9.195	1.75	1.273-2.227	0.243	7.196	-	-	-	-
Lu CW et al. [44]	8.07	-	0.79	-	4.61	-	0.75	-	4.38	-	0.61	-	-	-	-	-	4.34	-	0.31	-
Random	7.56	7.323-7.791	0.119	63.31	3.646	2.524-4.769	0.573	6.365	2.996	2.455-3.538	0.276	10.838	2.688	2.284-3.091	0.206	13.054	2.706	2.012-3.399	0.354	7.647
Prediction Interval	7.56	6.522-8.592			3.646	-0.49-7.782			2.996	0.583-5.41			2.688	1.217-4.159			2.706	0.038-5.373		
